# Advancements, Challenges and Prospects of Chemical Vapour Pressure at Atmospheric Pressure on Vanadium Dioxide Structures

**DOI:** 10.3390/ma11030384

**Published:** 2018-03-05

**Authors:** Charalampos Drosos, Dimitra Vernardou

**Affiliations:** 1 Delta Nano-Engineering Solutions Ltd., Paddock Wood, Kent TN12 6EL, UK; info@delta-nano.com; 2 Center of Materials Technology and Photonics, School of Applied Technology, Technological Educational Institute of Crete, 710 04 Heraklion, Crete, Greece; 3 Institute of Electronic Structure and Laser, Foundation for Research & Technology-Hellas, P.O. Box 1527, Vassilika Vouton, 711 10 Heraklion, Crete, Greece

**Keywords:** APCVD, VO_2_, processing parameters, 2D

## Abstract

Vanadium (IV) oxide (VO_2_) layers have received extensive interest for applications in smart windows to batteries and gas sensors due to the multi-phases of the oxide. Among the methods utilized for their growth, chemical vapour deposition is a technology that is proven to be industrially competitive because of its simplicity when performed at atmospheric pressure (APCVD). APCVD’s success has shown that it is possible to create tough and stable materials in which their stoichiometry may be precisely controlled. Initially, we give a brief overview of the basic processes taking place during this procedure. Then, we present recent progress on experimental procedures for isolating different polymorphs of VO_2_. We outline emerging techniques and processes that yield in optimum characteristics for potentially useful layers. Finally, we discuss the possibility to grow 2D VO_2_ by APCVD.

## 1. Chemical Vapour Deposition

### 1.1. General Information

CVD is a practical method of atomistic or near atomistic deposition having the ability to synthesize well-controlled dimensions and structures at reasonably low temperatures, high purity and in multiple formats such as single layer, multi-layer, composite and finally functional coatings. In its simplest incarnation, CVD encompasses a single precursor gas flowing into a chamber containing the substrate to be coated. Although, there are exceptions, the vapour of the reactive compound, usually an easily volatilized liquid or in some cases a solid, is sublimed directly and transported to the reaction zone by a carrier gas. A thin film is then deposited by chemical reaction or decomposition of the gas mixture on the substrate surface or in its vicinity at a defined temperature.

The precursors used within a variety of CVD techniques can be single source or dual source in origin. Single source precursors contain all the groups/elements required for successive thin film production. On the other hand, dual source precursors involve the interaction between multiple precursors for the synthesis of thin films. In each case, it is vital for production of thin films to deliver the gas phase precursors with a carrier gas. The most common carrier gases are N_2_, He or Ar, especially when highly reactive or pyrophoric reactants are used and in some cases, reactions entail an energy input from the carrier gas, e.g., H_2_ or O_2_ enrichment.

Reactor systems in CVD processes must allow controlled transport of the reactant and diluent gases to the reaction zone, maintain a defined substrate temperature and safely remove the gaseous by-products. These functions should be fulfilled with sufficient control and maximal effectiveness, which requires optimum engineering design and automation. The reactor in which the thin film deposition actually takes place is the essential part of the system and must be designed according to the specific chemical process parameters. To coat layers using Chemical Vapour Deposition at Atmospheric Pressure (APCVD), four basic types can be classified according to their gas flow and operation principles:

1. Horizontal tube displacement flow type.
2. Rotary vertical batch type.
3. Continuous—deposition type using premixed gas flow.
4. Continuous—deposition type employing separate gas streams.

### 1.2. CVD Processes

**Figure 1:**
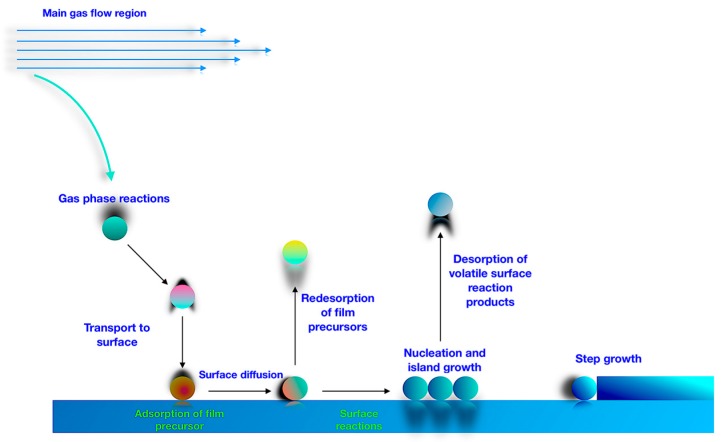
Schematic view of CVD (Chemical Vapour Deposition) process [1].

Any CVD process including APCVD involves the subsequent operations. First, the reacting gas is directed into the reactor. The gas moves towards its thermal equilibrium temperature and composition through gas-phase collisions and reactions. Near-equilibrated species are then transported to the reaction surface, the surface chemical reactions commence and the thin film is formed. The processes are summarized below (Figure 1) [1]:

1. Creation of active gaseous reactants.
2. Transport of the precursor to the CVD reactor.
3. Decomposition of gas phase precursor to remove gaseous by-products and grow reactive intermediates.
4. Gaseous reactants transportation onto substrate area.
5. Surface diffusion for nucleation and thin film growth.
6. Desorption of by-products and mass transport away from active reactive zone.

### 1.3. APCVD

**Figure 2:**
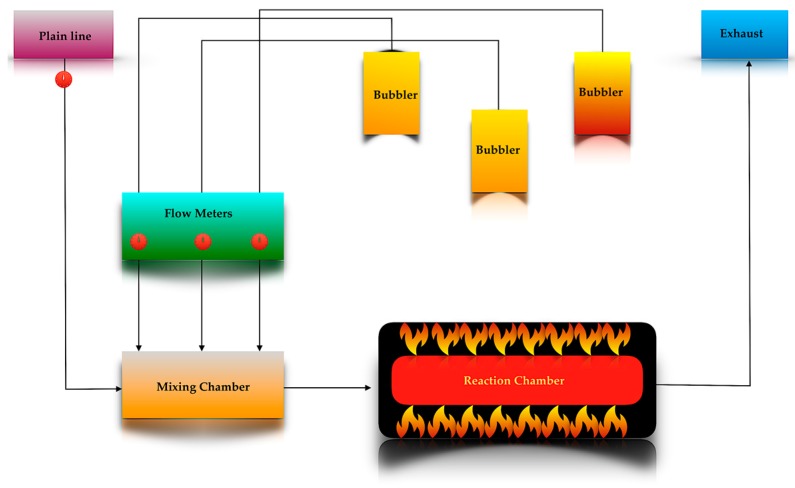
Schematic presentation of an APCVD (Chemical Vapour Deposition at Atmospheric Pressure) system [1].

A schematic presentation of the APCVD system is shown in Figure 2. It is designed with no joints in all outlet lines to avoid blocking. A flow of inert gas, usually nitrogen, is passed through the apparatus during all operations. The amount of the precursor delivered into the reactor is calculated from the Equation (1)

(1) $$a=\frac{\mathrm{VP}\times F}{\left(760-\mathrm{VP}\right)\times 24.4}$$

where a, is the amount of precursor (mol min^−1^), VP, is the vapour pressure of precursor at the bubbler’s temperature (mm Hg), F, is the nitrogen flow rate through the bubbler (L min^−1^) and 24.4, is a constant for the molar volume of an ideal gas at standard temperature and pressure (L mol^−1^).

In a typical APCVD experiment, once all temperatures are stabilized over time, the N_2_ is passed through the bubblers and then the precursor gas flow rate is directed into the mixing chamber where the mixture begins in order to be utilized before entering the reaction chamber for the deposition to take place. Once the allotted time is complete, the precursor bubbler is closed. The reactor heater is turned off and the substrate is allowed to cool down under an atmosphere of N_2_. Ideally, the carrier gas inlet flows should be fully saturated with precursor vapour; this can be achieved with knowledge of the precursor volatility and vapour pressure and then controlled by the carrier gas flow and bubbler temperature using flow meters and heating jackets.

## 2. Vanadium Oxides

The binary Vanadium-Oxygen phase diagram consists of a large number of phases between V_2_O_3_ and VO_2_ of the form V_n_O_2n−1_ commonly known as the Magneli phases [2] that exhibit distinctive electrical and optical properties. The variety of Vanadium-Oxygen stoichiometries emerges from the ability of vanadium atoms to adopt multiple oxidation states, which consequently results in synthetic challenges to control the structure of the materials [3].

More than ten kinds of crystalline phases of VO_2_ have been reported elsewhere, whereas some examples are monoclinic VO_2_ (M), tetragonal VO_2_ (R) and several metastable forms of VO_2_ (A), VO_2_ (B) and VO_2_ (C) [4]. Among these phases, only the rutile VO_2_ (R/M) phase undergoes a fully reversible metal insulator transition at a critical temperature (T_c_) [1], where an abrupt alteration in optical and electronic properties is observed making it ideal for optoelectronic switches [5], memristors [6], artificial neuron networks [7,8] and intelligent window coatings [9,10].

The high temperature phase (T > T_c_), has a tetragonal type structure characterized by chains of edge sharing [VO_6_] octahedral along the *c*-axis with equidistant vanadium atoms (V-V = 2.88 Å) [11]. While, the low temperature structure involves V^4+^-V^4+^ pairing with alternate shorter (0.265 nm) and longer (0.312 nm) V^4+^-V^4+^ distances along the *a*-axis and tilting with respect to the rutile *c*--axis [11]. At 25 °C, the lattice has unit cell parameters; *a* = 5.75 Å, *b* = 4.52 Å, *c* = 5.38 Å and *β* = 122.60° [12]. The lattice is the result of the distortion occurring at the high temperature metallic tetragonal phase.

The mechanism of metal insulator transition in VO_2_ has been investigated through computational, experimental and theoretical studies [13,14,15]. Nevertheless, the mechanism of the transition remains unresolved, since the VO_2_ phases exhibit diverse lattice structures but have analogous electronic properties.

## 3. Advancements

There have been numerous studies on the VO_2_ grown by APCVD since Maruyama and Ikuta utilized vanadium (III) acetylacetonate (V(acac)_3_) as a single-precursor to deposit polycrystalline pure VO_2_ films on fused quartz and sapphire single crystals [16]. In this review article, we will focus on the progress taking place during the last four years regarding the control of the processing parameters to isolate the VO_2_ phases strengthening the functional properties of APCVD VO_2_ layers.

**Figure 3:**
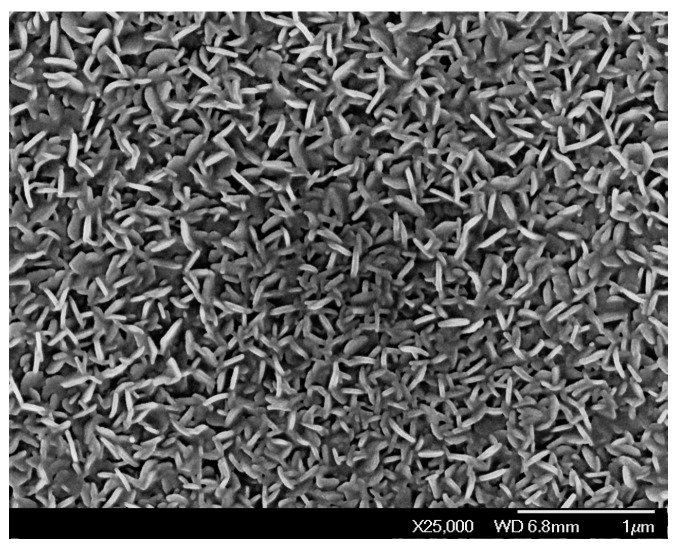
Field emission-scanning electron microscopy image of the APCVD tungsten doped VO_2_ coating.

**Figure 4:**
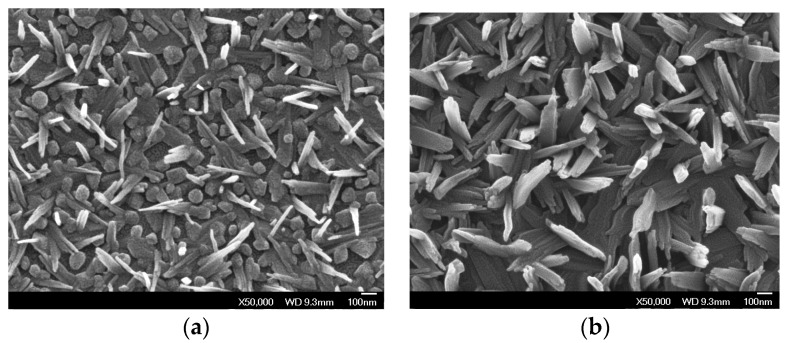
Field emission-scanning electron microscopy images of APCVD vanadium oxides using oxygen flow rate of 0.4 (**a**) and 0.8 L min^−1^ (**b**).

The growth of amorphous pure and tungsten doped VO_2_ coatings is possible on SnO_2_-precoated glass substrates using vanadyl (V) triisopropoxide (VO(OC_3_H_7_)_3_) as single-precursor [9,10,17,18]. It is interesting to note that the presence of tungsten in the lattice of VO_2_ changed the surface morphology to worm-like (Figure 3) from granular structure [9]. This approach has several advantages including the high vapour pressure of the precursor (i.e., decomposition over time and transport of unknown species are prevented). Additionally, the operations are simplified by removing the commonly necessary oxygen source, which is usually provided either in the form of pure gas or from an extra bubbler through H_2_O or alcohol. Vanadyl (IV) acetylacetonate (VO(acac)_2_) along with propanol, ethanol and O_2_ gas as oxygen sources is accomplished to grow VO_2_ of different crystalline orientations [19,20]. The *a*--axis textured monoclinic is enhanced with propanol and ethanol, while the 022-oriented single phase VO_2_ is obtained with O_2_ gas possessing grains (*a*--axis coatings) and agglomeration of grains forming rod-like structures (002-oriented phases). On controlling the oxygen gas flow rate (Figure 4), isolated monoclinic and metastable VO_2_ phases can also be achieved using VO(acac)_2_ as vanadium precursor on flexible [21] and SnO_2_-precoated glass substrates [22].

SnO_2_ was chosen as a substrate due to the similar crystalline structure with VO_2_, which can act as a template for the growth of rutile VO_2_ and promote the crystallinity of the oxide [23].

## 4. Challenges

**Figure 5:**
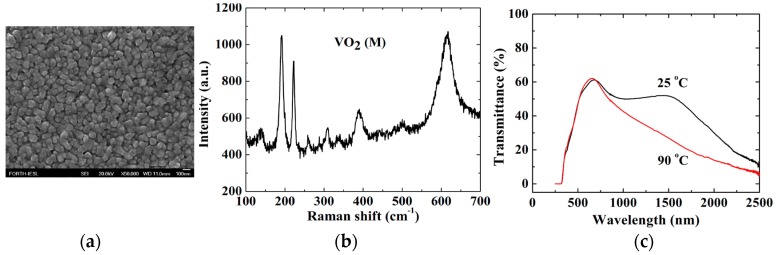
Field emission-scanning electron microscopy image (**a**) and (**b**) Raman spectroscopy of APCVD VO_2_ grown on SnO_2_-precoated glass substrates. (**c**) Transmittance spectra below T_c_ at 25 °C and above T_c_ at 90 °C over the region of 250–2500 nm to study the thermochromic performance.

**Figure 6:**
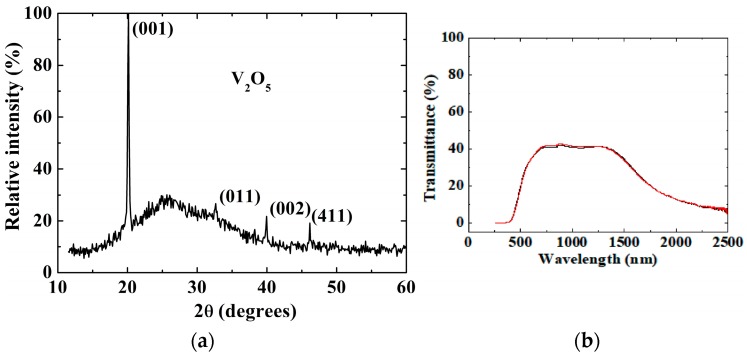
(**a**) X-ray diffraction of APCVD V_2_O_5_ grown on F-doped SnO_2_. (**b**) No change in transmittance spectra observed over the region 250–2500 nm below T_c_ at 25 °C (black colour) and above T_c_ at 90 °C (red colour).

A comparative study among VO(acac)_2_ and VCl_4_, the most utilized vanadium precursors for APCVD VO_2_, indicated that the transport rate of VO(acac)_2_ is lower than VCl_4_ [24]. This can be handled by increasing the temperature and the N_2_ flow rate in the bubbler. However, this is not anticipated because the precursor may decompose over time leading to irreproducible delivery rates and the transport of unknown species. On the other hand, VCl_4_ is highly reactive with H_2_O resulting in inhomogeneous films [25]. A new approach uses the ethyl acetate (EtAc) as an excellent oxygen precursor resulting in the precise control of the growth rate and porosity of the films after the optimization of VCl_4_/EtAc system [26]. A route to improve this system involves the combination of X-ray photoelectron spectroscopy (XPS) and X-ray absorption near-edge structure (XANES) to determine the effect of the substrate choice on the VO_2_ formation for functional properties such as thermochromism [27]. It is then possible to grow VO_2_ (Figure 5) onto substrates that induce lattice matching (SnO_2_) or others (F-doped SnO_2_) that promote a destabilization of V^4+^ ions and a further increase in V^5+^ deteriorating the functional properties (Figure 6).

Furthermore, monoclinic VO_2_ exhibits poor adhesion and is chemically susceptible to attack, restricting the use as solar control coating. In that respect, multi-functional, robust APCVD VO_2_/SiO_2_/TiO_2_ films on glass substrates demonstrates excellent solar modulation properties, high transparency and resistance to abrasion compared to single VO_2_ films of the same thickness [28]. 

## 5. Prospects and Outlook

In the field of APCVD VO_2_, the altering of the processing parameters and the manipulation of the substrate surface is just starting to be understood. New evolvements in experimental procedures such as the utilization of single vanadium precursor and the oxygen source have addressed APCVD routes in isolating the intrinsic material properties. There are numerous exciting challenges in developing VO_2_ with functional properties, which expand our understanding of the underlying chemistry and potentially lead to anticipated applications.

**Figure 7:**
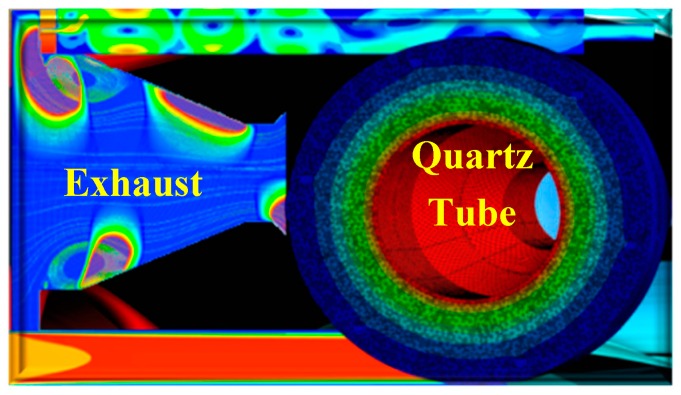
Simulation of transport species within the components of the APCVD reactor. The simulation showed an increase of the fluid’s velocity from 2.359 × 10^−3^ m s^−1^ to 3.283 × 10^−3^ m s^−1^, i.e., an increase of velocity of 39.18% due to temperature change. (Image courtesy of Delta Nano—Engineering Solutions Ltd., London, UK).

Two-dimensional (2D) VO_2_ can also be possible by APCVD through Computational Fluid Dynamics (CFD) simulations. CFD simulations are performed to evaluate and define the whole experimental process, before, while and after the experimental procedure isolating the intrinsic material properties (Figure 7). CFD results of exhaust and quartz tube presented the simulation procedure regarding the flow rates and the temperature distribution along the boundaries of the metallic parts. The flow rate of N_2_ was set at 0.1 L min^−1^ and the temperature in the inner boundaries was at 300 °C. Every aspect of the APCVD process is simulated to approach the optimal characteristics of the oxide in tandem to the surface to be deposited. Prospects in developing the growth of high-quality large-area materials with well-defined sizes, high dispersion and excellent control on layer thickness will then appear. The potential impact is illustrated by considering the exploitation possibilities of the high-performance materials by APCVD to create advanced devices for practical applications.
